# Identification of an AMPK Phosphorylation Site in Drosophila TSC2 (gigas) that Regulate Cell Growth

**DOI:** 10.3390/ijms16047015

**Published:** 2015-03-27

**Authors:** Myungjin Kim, Jun Hee Lee

**Affiliations:** Department of Molecular and Integrative Physiology, University of Michigan, Ann Arbor, MI 48109, USA; E-Mail: leeju@umich.edu

**Keywords:** Drosophila, AMPK, mTOR, mTORC1, TSC2

## Abstract

AMP-activated protein kinase (AMPK) is an important metabolic regulator that mediates cellular adaptation to diverse stresses. One of the AMPK substrates, tuberous sclerosis complex 2 (TSC2), was suggested to mediate AMPK-induced silencing of mTOR complex 1 (mTORC1) signaling that is critical for cell growth. However, it is not known whether the AMPK-dependent TSC2 phosphorylation, originally observed in mammalian cells, is conserved in invertebrates. Here we show that energy depletion inhibits mTORC1 signaling through the AMPK-TSC2 axis in Drosophila S2 cells. We have discovered an AMPK phosphorylation site in TSC2-like genes from many different invertebrate species including Drosophila. The site (Ser1338 in Drosophila TSC2) is specifically and efficiently phosphorylated by AMPK *in vitro*. To evaluate the functional role of this phosphorylation site *in vivo*, we generated transgenic flies that can express identical amount of either wild-type or phosphorylation-resistant mutant Drosophila TSC2 in a tissue-specific manner. In response to transgenic Sestrin induction, which causes ectopic AMPK activation and subsequent mTORC1 inhibition, wild-type Drosophila TSC2 synergistically reduced tissue growth in the dorsal epithelium of Drosophila wings. However, phosphorylation-resistant mutant Drosophila TSC2 was unable to show such a growth-inhibiting effect, suggesting that this phosphorylation is important for AMPK-dependent regulation of cell growth.

## 1. Introduction

AMP-activated protein kinase (AMPK) is a cellular energy sensor whose protein kinase activity is upregulated upon binding to AMP, which is accumulated when energy source has been depleted [1]. Once activated, AMPK regulates metabolism to adapt to the nutrient-scarce condition; AMPK upregulates catabolism while inhibiting anabolism. It is known that AMPK can control some of the key metabolic enzymes by altering its catalytic activity through direct phosphorylation [1]. However, AMPK can also alter the transcriptional program as well as cellular signaling pathways to produce more long-term adaptation of cells to the stringent environment.

Mechanistic target of rapamycin complex 1 (mTORC1) is another important nutrient-sensing protein kinase, which can respond to the level of various nutrients, including glucose and amino acids [2]. Unlike AMPK, mTORC1 is activated during nutritional abundance but is silenced upon starvation. Thus, once activated, mTORC1 regulates metabolism to adapt to the nutrient-rich environment; mTORC1 upregulates anabolism while inhibiting catabolism. For example, mTORC1 phosphorylates several translation regulators, including p70 ribosomal protein S6 kinase (S6K) and eukaryotic protein translation intiation factor 4E-binding protein (4E-BP) to boost synthesis of proteins [2]. In addition, mTORC1 upregulates lipid metabolism through activation of a lipogenic transcription factor SREBP [3] and nucleotide biosynthesis through phosphorylation of CAD [4,5], an enzyme that catalyzes the first three steps of *de novo* pyrimidine synthesis. On the other hand, mTORC1 suppresses autophagic catabolism by inhibiting an autophagy-initiating protein kinase ULK1/2 through direct phosphorylation [6] and by inhibiting lysosome biosynthesis through regulation of a transcription factor TFEB [7]. Thus, it is very important to understand how mTORC1 activity is physiologically regulated.

**Figure 1:**
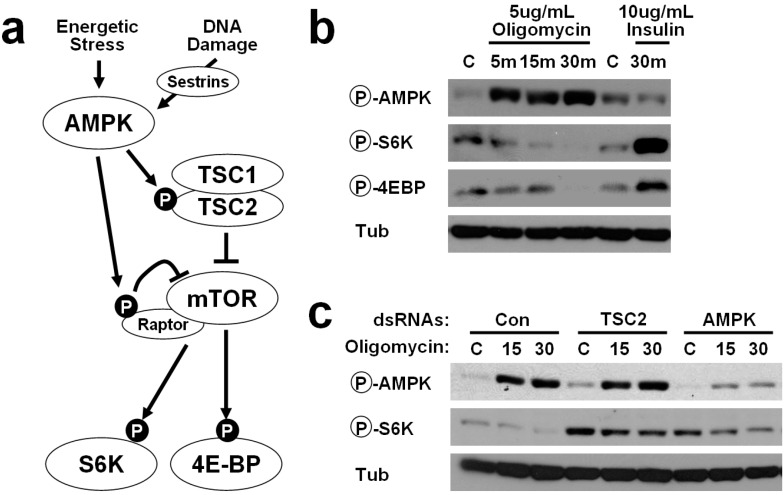
Energy depletion inhibits mTOR complex 1 (mTORC1) signaling through AMP-activated protein kinase (AMPK)-tuberous sclerosis complex (TSC) axis in *Drosophila* S2 cells. (**a**) Schematic representation of the relationship between AMPK and mTORC1 signaling; (**b**) Energy stress induced by oligomycin, a mitochondrial ATP synthase inhibitor, cause upregulation of AMPK phosphorylation, monitored by anti-phospho AMPK (Thr184, Cell Signaling) antibodies, and downregulation of TOR target gene phosphorylation, monitored by anti-phospho S6K (Thr398, Cell Signaling) and anti-phospho 4EBP (Thr37/Thr46, Cell Signaling) antibodies; (**c**) dsRNA-mediated silencing of TSC2 or AMPK suppressed energy stress-induced downregulation of TOR signaling activity. For detailed experimental conditions, see Experimental Section.

Although it has been repeatedly observed that mTORC1 is activated by amino acids, it is still poorly understood how mTORC1 biochemically senses amino acids [8]. Several molecules, such as a tRNA synthase [9] and a lysosomal amino acid transporter [10,11] are among the candidates which can mediate the sensing. In contrast, it has been relatively well characterized that mTORC1 responds to cellular energy levels through AMPK (Figure 1a). Tuberous sclerosis complex 2 (TSC2) is the first molecule suggested to link AMPK with mTORC1 regulation [12,13]. TSC2 is a GTPase-activating protein (GAP) for a GTPase named Rheb, which is critical for mTORC1 activation. Once activated, AMPK phosphorylates TSC2 at Ser1387, which causes its functional activation [12]. Activation of TSC2 leads to inactivation of Rheb, which subsequently results in silencing of the mTORC1 catalytic activity. However, because the residue corresponding to Ser1387 was not found in invertebrate homologues of TSC2 through conventional bioinformatic approaches, it was thought that this mechanism is not conserved in invertebrates [14]. In addition to TSC2, AMPK can phosphorylate Raptor, a regulatory subunit of mTORC1, through which AMPK can additionally downregulate catalytic activity of mTORC1 [15].

Here, by using cluster analysis for performing multiple sequence alignment between TSC2-like genes from various vertebrate and invertebrate species, we were able to identify an AMPK phosphorylation motif that is conserved throughout all the examined metazoan species. The target serine in this motif (Ser1338 in Drosophila TSC2) is corresponding to Ser1387 in human TSC2. Using Drosophila TSC2, which is also known as Gigas or dTSC2, we showed that AMPK can efficiently phosphorylate invertebrate homolog of TSC2 *in vitro*. Mutation of this phosphorylation site also diminished the function of TSC2 in suppressing mTORC1-dependent cell growth in Drosophila wing epithelium. Thus, our study shows that the signal transduction pathway composed of AMPK, TSC2 and mTORC1 is present in invertebrates including Drosophila.

## 2. Results and Discussion

### 2.1. Energetic Stress Inhibits mTORC1 Signaling in Drosophila Cells

Although it has been known that energy-dependent AMPK signaling can induce cell structure changes in both insect and mammalian cells [16], it is not clear whether it can regulate mTORC1 signaling in insect cells as in mammalian cells [12]. Thus, we treated Drosophila S2 cells with oligomycin, a blocker of mitochondrial ATP synthase, which can provoke ATP depletion and strong energetic stress. Oligomycin can also induce accumulation of reactive oxygen species [17], which is known to activate AMPK and inhibit mTORC1 in mammalian cells [18,19]. As expected, oligomycin induced strong activatory phosphorylation of AMPK at Thr184, which corresponds to Thr172 of human AMPK, confirming that oligomycin can induce AMPK activation in insect cells as in mammalian cells (Figure 1b). We have also examined phosphorylation of mTORC1 substrates, S6K and 4EBP, at Thr398 and Thr37/46, which can be induced upon 30 min of insulin stimulation (Figure 1b). Although oligomycin had very little effect on mTORC1-dependent phosphorylation of S6K and 4EBP at early time points (5 min), 30 min of oligomycin treatment almost completely eliminated both of the phosphorylation events (Figure 1b). These results suggest that energy depletion in Drosophila cells can block mTORC1 signaling.

### 2.2. AMPK-TSC2 Axis Links Energetic Stress and mTORC1 Regulation in Drosophila Cells

We tested whether AMPK and TSC2, two known mediators of energy-induced mTORC1 silencing in mammalian cells [12], are also critical for such process in Drosophila cells. Thus, we have silenced TSC2 and AMPK in Drosophila cells through dsRNA transfection. Silencing of either TSC2 or AMPK strongly increased the level of mTORC1-dependent S6K phosphorylation, and blunted the inhibition of S6K phosphorylation after 30 min of oligomycin treatement (Figure 1c). These results suggest that the AMPK-TSC2 axis links energetic stress and mTORC1 regulation also in Drosophila cells.

### 2.3. Identification of Putative AMPK Phosphorylation Sites in Drosophila TSC2

Although human and Drosophila TSC2 show significant homology to each other, pairwise sequence alignment between them shows that the region around Ser1387 in human TSC2 (the AMPK phosphorylation site) is very poorly conserved in Drosophila TSC2, as formerly reported [14]. Thus, it was originally determined that the Ser1387 site is not preserved between mammals and insects. However, after obtaining the result indicating that both AMPK and TSC2 are important for mTORC1 regulation in Drosophila S2 cells (Figure 1), we thought that more careful bioinformatic analysis may enable us to identify such site in Drosophila TSC2.

**Figure 2:**
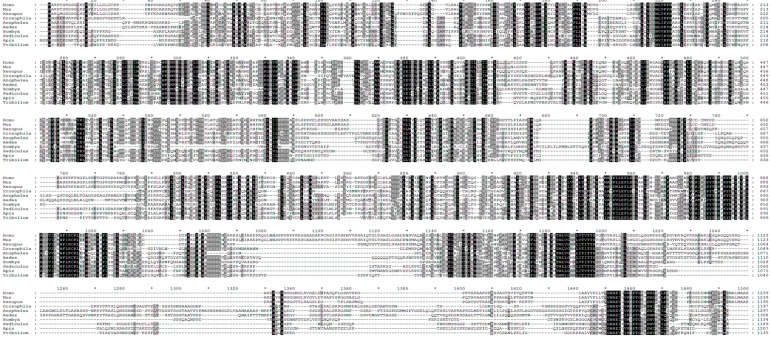

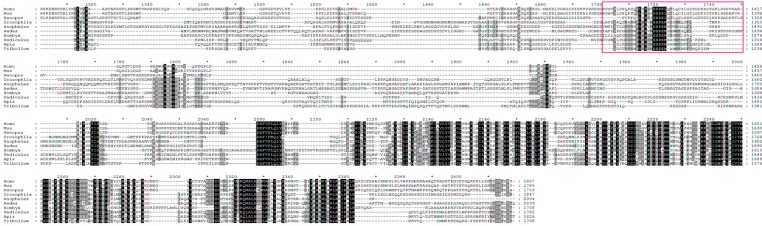
Multiple sequence alignment of tuberous sclerosis complex 2 (TSC2)-like proteins from different vertebrate and invertebrate species. Sequence alignment was conducted using CLUSTALW using TSC2 from the following animal species (from top to bottom): *Homo sapiens*, *Mus musculus*, *Xenopus tropicalis*, *Drosophila melanogaster*, *Anopheles gambiae*, *Aedes aegypti*, *Bombyx mori*, *Pediculus humanus corporis*, *Apis mellifera*, *Tribolium castaneum*. Pairwise alignment parameters are: Gap Open Penalty: 10.0, Gap Extension Penalty: 0.1, Weight Matrix: BLOSUM. Multiple alignment parameters are: Gap Open Penalty: 10.0, Gap Extension Penalty: 0.05, Weight Transition: YES (Value: 0.5), Hydrophilic Gaps: YES, Weight Matrix: ID (identity matrix). The alignment was constructed at GenomeNet (Kyoto University Bioinformatics Center) and rendered in GeneDoc v.2.7. Numbers and stars (*) above the sequences mark every 10 amino acids (a.a.). Residues were shaded according to their conservation between the examined species (black, 100%; dark grey, 80–99%; light grey, 60–79%). Red box highlights the conserved AMPK phosphorylation site, which is analyzed in more detail in Figure 3.

**Figure 3:**
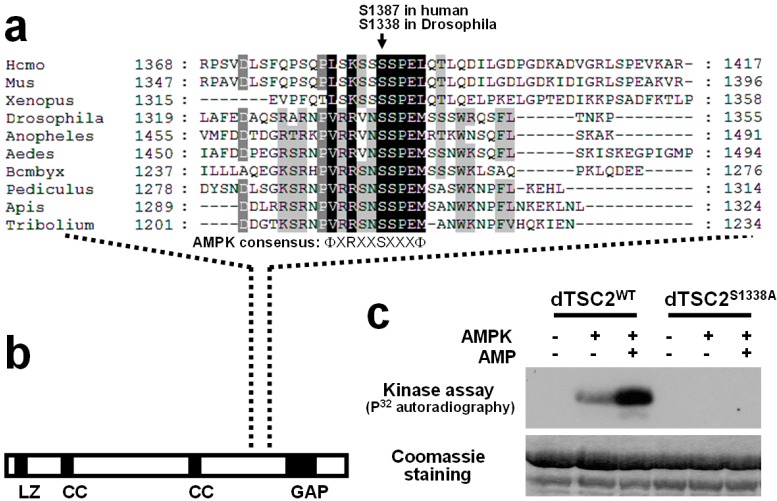
Conservation of AMPK phosphorylation site in Drosophila TSC2. (**a**) Multiple sequence alignment of TSC2-like proteins. A region surrounding the AMPK phosphorylation site (Ser1387 in human TSC2; highlighted in Figure 2 with red box) was magnified here. a.a. residues were shaded as in Figure 2; (**b**) Domain structure of Drosophila TSC2. The AMPK phosphorylation site falls between coiled coil (CC) and GAP domains; (**c**) *In vitro* kinase assay using purified AMPK protein and recombinant wild-type or Ser1338Ala-mutated Drosophila TSC2 protein corresponding to a.a. 1278-1371.

For this, we conducted two independent approaches. In the first approach, we have performed multiple sequence alignment of TSC2-like proteins from various vertebrate and invertebrate animals of distinct taxa. The rationale for this approach is that we would be able to minimize alignment errors or noises by comparing multiple sequences and identifying more functionally important residues that are widely and strictly conserved in different organisms. Interestingly, when we analyzed 10 different animal species for the multiple sequence alignment analysis, we were able to observe that the region surrounding Ser1387 is significantly conserved among the TSC2 proteins (Figure 2). In this alignment, Ser1338 in Drosophila TSC2 corresponds to the Ser1387 in human TSC2, and the sequence around these serines is very highly conserved throughout the species (Figure 3a). This region is located between highly conserved coiled coil (CC) and GAP domains of TSC2 (Figures 2 and Figure 3b). The second approach is to find the AMPK phosphorylation consensus motif (ФXRXXSXXXФ, Ф refers to hydrophobic residues) in Drosophila TSC2. Interestingly, the only site perfectly matching the AMPK motif in Drosophila TSC2 corresponds to the evolutionarily conserved region identified from the multiple sequence alignment (Figure 3a).

### 2.4. Ser1338 in Drosophila TSC2 Is Phosphorylated by AMPK

We were curious whether this putative phosphorylation site (Ser1338 in Drosophila TSC2) can be indeed phosphorylated by AMPK. Thus, we have set up an *in vitro* kinase assay using a recombinant Drosophila TSC2 protein purified from *E. coli* and an active AMPK holoenzyme purified from rat liver. Using this assay, we have found that Drosophila TSC2 is efficiently phosphorylated by AMPK, which was further activated by addition of AMP, an AMPK activator (Figure 3c). In contrast, alanine substitution of Ser1338 (Ser1338Ala) in the Drosophila TSC2 protein completely abolished the *in vitro* phosphorylation event mediated by AMPK (Figure 3c), indicating that Ser1338 of Drosophila TSC2 is indeed an AMPK phosphorylation site.

### 2.5. Ser1338 in Drosophila TSC2 Is Necessary for Its Growth-Regulating Function

**Figure 4:**
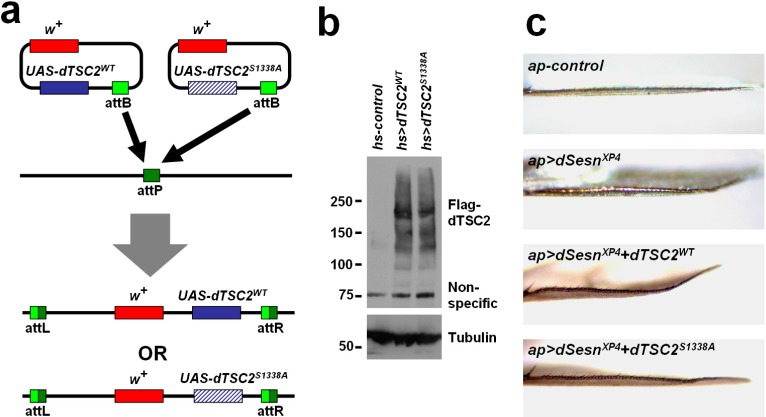
Ser1338 is required for genetic interaction between Drosophila TSC2 and Sestrin. (**a**) Schematic representation of how Drosophila TSC2 transgenic flies were made. Plasmid which can express wild-type or Ser1338Ala-mutated Drosophila TSC2 was inserted into an identical genomic location (the attP site) through phiC31-mediated recombination; (**b**) Heat shock (hs) Gal4-mediated induction of Drosophila TSC2 proteins. Adult flies of indicated genotypes were incubated overnight at 30 °C and subjected to immunoblot analysis of indicated proteins. Molecular weight markers are in kDa; (**c**) Wing blades of the flies expressing indicated transgenic elements were imaged under a light dissection microscope. *dSesn^XP4^* is an EP allele of *dSesn* that can drive tissue-specific Sestrin overexpression [23].

TSC2 is an important tumor suppressor whose loss can induce hyperplastic cell growth due to mTORC1 overactivation [20]. Overexpression of TSC2, when combined with other signaling components in the same pathway, can lead to mTORC1 inhibition and substantial reduction of cell growth. To examine the role of Ser1338 in cell growth regulation, we have generated transgenic flies which can express wild-type and Ser1338Ala-mutated Drosophila TSC2 from an identical genomic location through phiC31-att system (Figure 4a). We have confirmed that both forms of TSC2 were efficiently expressed in Drosophila tissues at a comparable level (Figure 4b). However, as formerly reported [20], expression of TSC2 alone did not cause any substantial alterations in cell growth on its own (data not shown).

It has been recently shown that Sestrin-family proteins can inhibit mTORC1-dependent cell growth through the AMPK-TSC2 axis [21,22,23] as well as through the GATOR-Rag axis [24,25,26]. When overexpressed in Drosophila dorsal wing epithelium, Sestrin specifically reduces growth of the target compartment, resulting in a slight bent-up wing phenotype (Figure 4c). Because Sestrin and Drosophila TSC2 are under the same genetic pathway, we have tested whether these components can synergistically reduce tissue growth in this system. Indeed, simultaneous expression of Sestrin and wild-type Drosophila TSC2 induces much more pronounced bent-up wing phenotype compared to the phenotype induced by Sestrin expression alone (Figure 4c). Interestingly, Ser1338Ala-mutated Drosophila TSC2 was unable to produce such synergistic genetic interaction (Figure 4c). Considering that Sestrin induces strong AMPK activation in both Drosophila and mammalian tissues [21,22,23], our results indicate that AMPK-dependent Ser1338 phosphorylation of Drosophila TSC2 is indeed important for growth regulation during Drosophila wing development.

## 3. Experimental Section

### 3.1. Cell Culture

Drosophila S2 cells are from the Drosophila Genomics Resource Center (DGRC) and cultured in Schneider’s medium plus 10% FBS and Peni-strep, according to instructions from the Drosophila RNAi Screening Center (DRSC, www.flyrnai.org). Oligomycin and insulin were purchased from Sigma (St. Louis, MO, USA). dsRNAs against *white* (control), *TSC2* and *AMPK* were generated and treated as recommended by DRSC, and corresponds to DRSC30065, DRSC29781 and DRSC28055 amplicons.

### 3.2. Immunoblotting

Cells and tissues were lysed in cell lysis buffer (20 mM Tris-Cl pH 7.5, 150 mM NaCl, 1 mM EDTA, 1 mM EGTA, 2.5 mM sodium pyrophosphate, 1 mM β-glycerophosphate, 1 mM Na_3_VO_4_, 1% Triton-X-100) containing protease inhibitor cocktail (Roche, Penzberg, Upper Bavaria, Germany), and processed as formerly described [27]. Protein samples were boiled in SDS sample buffer for 5 min, separated by SDS-PAGE, transferred to PVDF membranes and probed with primary antibodies (1:200 for Santa Cruz Antibodies, and 1:1000 for all other antibodies). After incubation with secondary antibodies conjugated with HRP (Bio-rad, Hercules, CA, USA; 1:2000), chemiluminescence was detected using X-ray films (Phenix, Candler, NC, USA) or LAS4000 (GE, Fairfield, CT, USA) systems.

### 3.3. Antibodies

Phospho-Thr172 AMPK and phospho-Thr37/46 4EBP antibodies from Cell Signaling (Danvers, MA, USA) were formerly shown to cross-react with phospho-Thr184 Drosophila AMPK and phospho-Thr37/46 Drosophila 4EBP [16,28]. Phospho-Thr398 Drosophila S6K antibody is also from Cell Signaling. Flag monoclonal antibody (M2) is from Sigma. Tubulin monoclonal antibody is from Developmental Studies Hybridoma Bank (DSHB, Iowa City, IA, USA). 

### 3.4. In Vitro AMPK Kinase Assay

As formerly described [16], the protein kinase assay was performed in a solution consisting of HEPES-Brij Buffer, 0.2 mM ATP (with 0.5 mCi/mL γ-^32^P-ATP for radioactive assay), and 1 mg of protein substrate at 30 °C for 20 min, with or without 0.3 mM AMP. Active AMPK holoenzyme was purchased from Upstate (14-305). GST-tagged recombinant dTSC2 proteins, purified from BL21 *E. coli* transformed with pGEX 4T-1 vector expressing dTSC2 (a.a. 1278-1371), were used as substrates. The assay samples were subjected to SDS–PAGE, coomassie staining and autoradiography.

### 3.5. Drosophila Strains and Culture

Wild-type dTSC2-coding sequence was derived from LD36178 cDNA (Berkeley Drosophila Genome Project). Ser1338Ala mutation was introduced to dTSC2 through a PCR-based site-directed mutagenesis. Both forms of dTSC2 was cloned into a pUAST-attB vector [29] and fully sequenced. pUAST-attB-dTSC2^WT^ and pUAST-attB-dTSC2^S1338A^ were microinjected into y^1^ M{vas-int.Dm}ZH-2A w^*^; *M{3xP3-RFP.attP'}ZH-22A* flies and stable transformants were isolated by presence of *mini-white^+^* marker. *hs-Gal4*, *ap-Gal4* and *dSesn^XP4^* were formerly described [23]. 

## 4. Conclusions

We have shown that energy deprivation in Drosophila cells can provoke mTORC1 inhibition in an AMPK- and TSC2-dependent manner. In addition, we have revealed that Drosophila TSC2 has an authentic AMPK phosphorylation site, which plays a key role for tissue growth-regulatory function of the protein. These data indicate that the AMPK-TSC2 signaling axis, originally discovered in mammalian cells, is highly conserved in invetebrate systems such as in Drosophila, and is critical for energy- and stress-dependent modulation of cell and tissue growth.
